# Syndrome de Lynch: à propos d'un cas et revue de la litterature

**DOI:** 10.11604/pamj.2016.24.142.4398

**Published:** 2016-06-14

**Authors:** Laila Bouguenouch, Imane Samri, Khadija Belhassan, Hanane Sayel, Meriame Abbassi, Sanae Bennis, Dafr Allah Benajah, Adil Ibrahimi, Afaf Amarti, Karim Ouldim

**Affiliations:** 1 Unité de Génétique Médicale et d'Oncogénétique, Laboratoire Central d'Analyses Médicales CHU Hassan II, Fès, Maroc; 2 Service d'Anatomie-Pathologique, Laboratoire Central d'Analyses Médicales CHU Hassan II, Fès, Maroc; 3 Service de Gastro-entérologie, CHU Hassan II, Fès, Maroc

**Keywords:** Syndrome de Lynch, gène MLH1, syndrome HNPCC, oncogénétique, Lynch syndrome, HNPCC syndrom, oncological

## Abstract

Le syndrome de Lynch, ou cancer colorectal héréditaire sans polypose ou HNPCC (hereditary non-polyposis colorectal cancer), est la forme la plus fréquente de cancer colorectal héréditaire. Il conduit à une augmentation de la susceptibilité à développer des cancers, au premier rang le cancer colorectal, le cancer de l'endomètre chez les femmes, et dans une moindre mesure, d'autres cancers (ovaire, intestin grêle, estomac, voies excrétrices urinaires et hépatobiliaires). Ainsi, le risque cumulé de développer un cancer colorectal ou de l'endomètre à l’âge de 80 ans s’élève respectivement à 20 et 40%. Ces cancers sont caractérisés par leur contexte d'atteinte familiale, leur survenue à un âge précoce, ainsi que par le développement de cancers métachrones chez un même individu. Ce syndrome se transmet de manière autosomique dominante. Les gènes dont l'altération est associée à l'existence d'un syndrome HNPCC appartiennent à la famille des gènes de réparation des mésappariements de l'ADN (DNA mismatch repair ou MMR): MSH2, MLH1 et MSH6 sont impliqués, par ordre décroissant de fréquence, dans respectivement 35%, 25% et 2% des cas. Une surveillance coloscopique et gynécologique est proposée aux personnes porteuses d'une mutation constitutionnelle du gène MSH2, MLH1 ou MSH6. Nous rapportons une des premières observations marocaines d'un syndrome de Lynch dont la mutation constitutionnelle du gène MLH1 a été identifiée chez un des membres de la famille atteint d'un cancer du côlon. Suite à la demande d'autres sujets sains de la même famille, un diagnostic presymptomatique a été effectué conduisant à une stratégie de surveillance adaptée. A travers notre observation nous illustrons le rôle de l'oncogénétique dans la prise en charge des patients cancéreux et de leurs familles.

## Abstract (en)

Lynch syndrome or hereditary nonpolyposis colorectal cancer (HNPCC) is the most common form of hereditary colorectal cancers. It increases cancer susceptibility, the risk of colorectal cancer in first-degree, endometrial cancer in women, and to a lesser extent, other cancers (ovarian, small bowel, stomach, urinary tract and hepatobiliary). Thus, the cumulative risk of developing colorectal cancer or endometrial cancer at the age of 80 years rises to 20 and 40% respectively. These cancers are characterized by a positive family history, their occurrence at an early age, and by the development of metachronous cancers in the same individual. This syndrome is transmitted in an autosomal dominant manner. The genes whose alteration is associated with the presence of an HNPCC belong to the family of DNA mismatch repair genes (DNA mismatch repair or MMR): MSH2, MLH1, and MSH6 are involved, in decreasing order of frequency, in 35%, 25% and 2% of cases respectively. Colonoscopic and gynecological monitoring is recommended for patients with a constitutional mutation in MSH2, MLH1 or Msh6 genes. We report one of the first moroccan case with Lynch syndrome whose constitutional mutation in the MLH1 gene was identified in a family member with colon cancer. In reply to the inquiry ofother healthy family members, a presymptomatic diagnosis was made allowing to formulate an appropriate monitoring strategy. Our study aims to highlight the role of oncogenetics in the management of patients with cancer and their families.

## Introduction

La cancérogenèse colorectale est étudiée par de nombreuses équipes et l’évolution des connaissances peut entraîner des changements dans les stratégies de prise en charge des patients [1]. Le cancer est l’émergence d'un clone cellulaire qui prolifère, envahit, métastase malgré les différents niveaux de contrôle de l'organisme. Ceci n'est possible que par l'accumulation de nombreuses anomalies génétiques, suivant différentes voies de cancérogenèse. Le cancer colorectal (CCR) est un des meilleurs exemples de ce processus multi étape de cancérogenèse [2]. Les gènes touchés par ces anomalies, ou gènes cibles, sont nombreux: ce sont potentiellement tous les gènes qui contrôlent le cycle cellulaire, l'apoptose, la migration des cellules et tous les phénomènes de la cancérogenèse. Globalement, ils sont regroupés dans deux catégories: les oncogènes et les gènes suppresseurs de tumeur [3].

Le syndrome HNPCC (syndrome de Lynch) est responsable de 3 à 5% des cancers colorectaux [4]. De transmission autosomique dominante, il est dû à une mutation constitutionnelle d'un des gènes du MMR, essentiellement MLH1 et MSH2. Les tumeurs du syndrome HNPCC présentent une instabilité des microsatellites (phénotype MSI). Le spectre tumoral du syndrome HNPCC est large, mais les risques tumoraux majeurs sont le CCR (risque cumulé de 80% à 80 ans) et le cancer de l'endomètre (42 à 60% à 70 ans) [2].

Nous rapportons une observation d'une famille, présentant les critères du syndrome de Lynch est chez qui la mutation du gène MLH1 a été identifié chez l'un des patients atteints d'un cancer du côlon. Dans le cadre du suivi de cette famille prédisposée au spectre cancérologique du syndrome de Lynch un diagnostic présymptomatique a été fait à la demande des autres membres de la famille. Ce test nous a permis d’élaborer un processus de surveillance selon les recommandations internationales du syndrome de Lynch.

## Patient et observation

**Figure 1:**
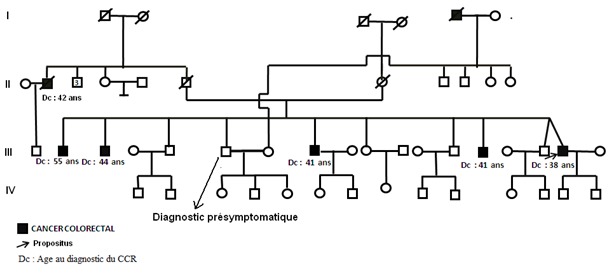
Arbre généalogique

Suite à une consultation d'oncogénétique du propositus (patient de 38 ans qui présente un adenocarcinome du colon), appartenant à une famille avec plusieurs cas de cancer du côlon (arbre généalogique, Figure 1), après consentement éclairé, nous avons indiqué les démarches suivantes: l’étude d'expression des protéines de réparation des mésappariements de l'ADN par immunohistochimie; la recherche d'instabilité micro satellitaire sur tumeur; la recherche d'une mutation germinale des gènes du système MMR à partir de l'ADN lympocytaire, selon les résultats de l'immunohistochimie et du profil MSI (microsatellites instability (MSI)).

Les résultats montrent un défaut d'expression des protéines MLH1 et PMS2 et une instabilité microsatellitaire. Suite à ces données et celui de l'arbre généalogique, qui orientent vers le syndrome de Lynch, la recherche d'une mutation du gène MLH1 est indiquée par séquençage directe (analyse de l'ADN génomique par séquençage (*Dye Terminator, ABI PRISM 3130*)) L’étude moléculaire a mis en évidence chez notre patient la présence d'une mutation à l’état hétérozygote du gène MLH1: c.T > C, p.Met1. Suite à la demande de son frère, âgé de 36 ans, une étude moléculaire a conclue qu'il est porteur de la même mutation.

Le protocole de prise en charge des sujets porteurs de la mutation MLH1, est mis en œuvre dans un cadre de prise en charge multidisciplinaire (colonoscopie…). Le compte rendu de la dernière colonoscopie a décrit un aspect de polypose prédominante au niveau du colon proximal faite d'un grand nombre de petits polypes de taille inférieure à 1 cm.

## Discussion

Il est important de reconnaître les formes héréditaires de CCR pour pouvoir dépister et surveiller la famille du cas index, et pour pouvoir adapter la thérapeutique [5]. Devant un cancer colique, un cancer héréditaire doit être évoqué s'il survient avant 50 ans, surtout au niveau du colon droit, et/ou avec une histologie évocatrice (contingent mucineux, infiltrat lymphocytaire), surtout s'il est associé, chez le patient ou ses apparentés, à une tumeur du spectre HNPCC. L'instabilité des microsatellites doit alors être recherchée dans la tumeur. Plusieurs critères ont été proposés pour guider cette recherche: les critères de Bethesda révisés sont les plus récents [6].

Ces familles doivent se voir proposer une consultation d'oncogénétique. La surveillance porte sur les apparentés porteurs de la mutation ou sur les sujets à risque si aucune mutation n'est retrouvée [2].

Le syndrome de Lynch ou cancer colorectal héréditaire sans polypose ou HNPCC (hereditary non-polyposis colorectal cancer), est une maladie héréditaire autosomique dominante, en rapport avec une mutation germinale de l'un des gènes de réparation des mésappariements de l'ADN, il représente la forme la plus fréquente de cancer colorectal héréditaire. Son diagnostic repose sur la réunion de trois critères, définis en 1991 à Amsterdam, et revus en 1999: (i) au moins 3 sujets atteints de cancers appartenant au spectre étroit du syndrome HNPCC (cancers colorectaux, cancers de l′endomètre, de l´intestin grêle, des voies urinaires) et histologiquement prouvés, (ii) unis 2 à 2 par un lien de parenté au premier degré sur 2 générations, (iii) un des cancers au moins s´étant révélé avant l´âge de 50 ans [7].

Cette anomalie conduit à une augmentation de la susceptibilité à développer des cancers, au premier rang le cancer colorectal sans polypose (Human nonpolyposis colorectal cancer (HNPCC)), le cancer de l'endomètre chez les femmes, et dans une moindre mesure, le cancer de l'ovaire, de l'intestin grêle, de l'estomac, des voies excrétrices urinaires et hépatobiliaires (Tableau 1). Ainsi, le risque cumulé de développer un cancer colorectal ou de l'endomètre à l’âge de 80 ans s’élève respectivement à 20 et 40% [8]. Les gènes dont l´altération est associée à l´existence d´un syndrome HNPCC appartiennent à la famille des gènes de réparation des mésappariements de l´ADN (DNA mismatch repair ou MMR), autrement dit dans le contrôle de la fidélité de la réplication: MSH2, MLH1 et MSH6 sont impliqués, par ordre décroissant de fréquence, dans respectivement 35%, 25% et 2% des cas [9]. Ces cancers sont caractérisés par leur contexte d'atteinte familiale, leur survenue à un âge précoce, ainsi que par le développement de cancers métachrones chez un même individu. Comme pour la plupart des cancers, le diagnostic précoce de ces tumeurs bouleverse leur pronostic. Par conséquent, le dépistage apparait crucial chez ces patients prédisposés, grâce à un suivi régulier et adapté [1].

**Tableau 1:** Risques de cancer chez les individus porteurs d'une mutation constitutionnel à l’état hétérozygote des gènes MLH1 et MSH2 (syndrome Lynch), avant l'age de 70 ans, comparativement à la population générale

Type de cancer	Risque dans la population général	Syndrome de Lynch (mutation à l’état hétérozygote MLH1 et MSH2)
		Risque
Colon	5.5%	52%-82%
Endomètre	2.7%	25%-60%
Estomac	<1%	6%-13%
Ovaire	1.6%	4%-12%
Voies biliaires	<1%	1.4%-4%%
Voies urinaires	<1%	1%-4%
Intestin grêle	<1%	3%-6%
Cerveau, Système nerveux central	<1%	1%-3%

Nous rapportons l'un des premiers diagnostics presymptomatique d'une famille marocaine porteuse d'une mutation germinale du gène MLH1, qui présente plusieurs cas de cancers de colon. En effet l’étude moléculaire a mis en évidence chez notre patient porteur du cancer du côlon, la présence d'une mutation à l’état hétérozygote du gène MLH1: c.T > C, p.Met1. Suite à la demande de son frère, âgé de 36 ans, et au cours d'une consultation d'oncogénétique et après son consentement éclairé, une étude moléculaire a conclu qu'il porteur de la même mutation. Le compte rendu de la colonoscopie a décrit un aspect de polypose prédominante au niveau du colon proximal faite d'un grand nombre de petits polypes de taille inférieure à 1 cm. Cet aspect endoscopique impose l'existence d'un autre facteur génétique de prédisposition et justifie d'explorer le gène *MUTYH* pour notre sujet. Selon les recommandations du comité d'oncogénétique de l'INCa (Institut National du Cancer Français), les personnes porteuses d'une mutation d'un gène MMR présentent un risque élevé de développer un cancer colorectal et de l'endomètre. Il existe également d'autres risques tumoraux, beaucoup plus faibles, associés à ces mutations. La prise en charge et le suivi du risque colorectal doit débuter dès l’âge de 20 ans. La surveillance doit être effectuée par endoscopie colorectale complète avec chromoscopie par indigo-carmin, réalisée tous les 2 ans, en insistant sur la bonne préparation colique. La chirurgie prophylactique colorectale sur côlon sain n'est pas recommandée. Dans le cas d'un cancer diagnostiqué, le choix entre colectomie segmentaire ou colectomie subtotale avec anastomose iléorectale doit être discuté en fonction de l’âge du patient et de son souhait.

La découverte d'un cancer lié à HNPCC peut modifier l'indication chirurgicale. En effet, le risque de nouveau cancer colique étant de 55% à 20 ans, la colectomie totale doit être discutée en cas de cancer curable [9]. L'hystérectomie non conservatrice préventive peut y être associée pour les femmes ménopausées. La colectomie prophylactique est en revanche l'objet de controverses [10]. Dans ce cadre, la surveillance par coloscopie a montré une réduction de 63% du risque de cancer colorectal, ainsi qu'une réduction de la mortalité [11]. Les recommandations actuelles reposent sur la réalisation d'une coloscopie tous les deux ans à partir de l’âge de 20 ans [6]. Concernant le dépistage gynécologique, la littérature fournit très peu de données sur son intérêt. Si le bénéfice n'est pas prouvé [1], le dépistage gynécologique est néanmoins proposé. La plupart des recommandations internationales propose un dépistage annuel, comportant un examen clinique, une échographie et un prélévement endométrial [12]. La surveillance gastrique repose sur la réalisation d'une fibroscopie oesogastroduodénale avec recherche d'Helicobacter pylori, à l'occasion de la première coloscopie. La surveillance ovarienne passe par l’échographie endovaginale. Le dépistage systématique des autres tumeurs n'est actuellement pas recommandé, au regard du faible niveau de sur-risque et de l'absence de consensus pour les modalités de surveillance. Le syndrome de Lynch prédispose à de multiples tumeurs, impliquant dès lors plusieurs disciplines médicales. Deux points apparaissent essentiels: d'une part, plutôt qu'un suivi isolé par chacun des spécialistes, une prise en charge conjointe et coordonnée apparait souhaitable tant d'un point de vue médical, pour l'instauration d'un plan de dépistage adapté, que d'un point de vue humain, en limitant la multiplicité des consultations. D'autre part, la prise en charge optimale de cette pathologie rare relève préférentiellement d'un regroupement de l'ensemble des intervenants, associant chirurgiens gynécologues oncologues, hépatogastroentérologues, chirurgiens viscéraux, chirurgiens urologues, oncogéneticiens, radiologues et psychologues.

## Conclusion

L'identification de gènes de prédisposition au cancer a permis l'introduction de nouvelles analyses génétiques destinées aux personnes dont les antécédents médicaux personnels et/ou familiaux sont évocateurs d'une forme héréditaire de cancer [13]. Le syndrome HNPCC (Hereditary Non-Polyposis Colorectal Cancer)/Lynch est une forme héréditaire non polyposique de cancers colorectaux, pourvoyeuse de 3% environ de l'ensemble des cancers colorectaux. Il est également associé à une augmentation significative du risque d'autres types tumoraux et notamment de cancer de l'endomètre [14]. Le diagnostic du syndrome HNPCC/Lynch repose actuellement sur l'identification de la mutation causale. A travers cet article nous insistons sur le rôle de l'oncogénétique dans la prise en charge du cancer au Maroc, ouvrant l’ère de la médecine prédictive dans le domaine de la cancérologie.
